# Can Optogenetic Tools Determine the Importance of Temporal Codes to Sensory Information Processing in the Brain?

**DOI:** 10.3389/fnsys.2015.00174

**Published:** 2015-12-21

**Authors:** Gytis Baranauskas

**Affiliations:** Neurophysiology Laboratory, Neuroscience Institute, Lithuanian University of Health SciencesKaunas, Lithuania

**Keywords:** temporal coding, rate coding, sensory inputs, information, optogenetics, channelrhodopsin, visual system, olfactory system

## Abstract

There is no doubt that optogenetic tools caused a paradigm shift in many fields of neuroscience. These tools enable rapid and reversible intervention with a specific neuronal circuit and then the impact on the remaining circuit and/or behavior can be studied. However, so far the ability of these optogenetic tools to interfere with neuronal signal transmission in the time scale of milliseconds has been used much less frequently although they may help to answer a fundamental question of neuroscience: how important temporal codes are to information processing in the brain. This perspective paper examines why optogenetic tools were used so little to perturb or imitate temporal codes. Although some technical limitations do exist, there is a clear need for a systems approach. More research about action potential pattern formation by interactions between several brain areas is necessary in order to exploit the full potential of optogenetic methods in probing temporal codes.

It’s quite likely that in neurons the main information unit is an action potential. To a certain extent, this conclusion follows from the first experiments indicating that the shape of action potentials is rather stereotypical and does not depend much on the stimulus (Adrian and Zotterman, 1926b). Thus, other factors such as rate and timing of action potential occurrence encode the features of sensory stimuli. Rate codes convert stimulus parameters into the number of action potentials fired during a specified time interval (**Figure 1**). Temporal codes convey sensory input information through specific spike times, either of a single neuron or a neuronal population. In the simplest form of temporal coding the degree of coincidence or synchrony but not the overall number of action potentials carries information about sensory inputs (**Figure 1**). However, other temporal characteristics of spike sequences such as burst number, variance of spike frequency or any reproducible sequence of time intervals between action potentials may encode stimulus features (Izhikevich, 2006; Kostal et al., 2007).

**FIGURE 1:**
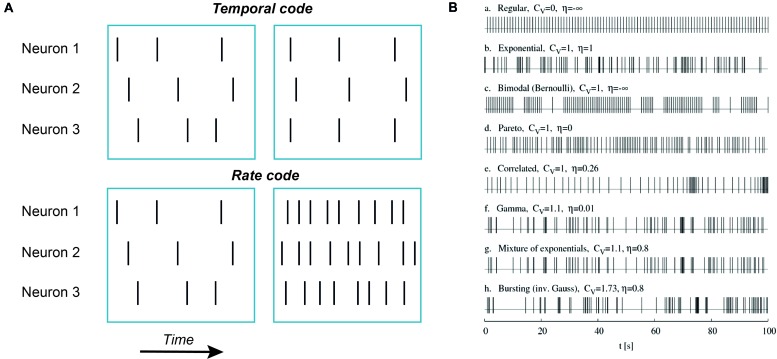
**Examples of temporal codes. (A)** Schematic representation of rate and temporal codes. Each vertical bar represents a single action potential. Top row: an example of temporal coding by three neurons. Note that there is no difference in action potential numbers between left and right blue boxes for all three neurons but on the right action potentials are aligned in time between the first and the third neuron while in the second neuron action potentials are delayed and fired after a constant time interval. Such an alignment in time (coincidence) or a constant time delay can be detected by neurons. Bottom row: an example of rate coding. No neuron fires action potentials in synchrony with other neurons, only the number of action potentials is increased. **(B)** Simulated trains of action potentials of the same mean firing frequency but different variance and randomness. Potentially, all these action potential patterns can represent different temporal codes. Adapted with permission from Kostal et al. (2007).

In general, there is no strict distinction between rate and temporal codes. In spite of attempts to have a rigorous definition for codes (Theunissen and Miller, 1995), the distinction between a temporal and a rate code will depend on the duration of the encoding time window. Any change in rate is associated with a change in the time interval between action potentials that can be viewed as a change in the temporal code. Similarly, any change in the inter-spike time interval duration, a hallmark of temporal coding, can be viewed as an instantaneous rate change.

There is a long history of published papers presenting arguments in favor of either rate or temporal coding. It is beyond the scope of this paper to review this topic. It can be noted that indirect evidence for both rate and temporal codes can be found in the brain. While the rate code hypothesis is supported by numerous studies demonstrating correlation between the action potential rate and the stimulus intensity (Adrian and Zotterman, 1926a,b; Hartline and Graham, 1932; Shadlen and Newsome, 1994), temporal code supporters can present a large number of phase locked responses, when the time of action potential occurrence is strongly correlated with either the stimulus onset or the phase of the neuronal population oscillations (Erulkar, 1959; Gerstein et al., 1968; McClurkin et al., 1991; Ainsworth et al., 2012; Royer et al., 2012; Buzsaki and Moser, 2013). However, the main question, in which form information is transmitted from one population of neurons to another, remains unanswered in most of the cases because all the above mentioned observations rely on correlation between the action potential times and the stimulus and no causality can be established.

Optogenetic tools appear ideally suited for temporal code probing. Light sensitive channels enable rapid control of neuronal activity: action potentials can be evoked with nearly millisecond precision. Meanwhile, genetic methods enable the delivery of light sensitive channelrhodopsins to specific sets of neurons (Deisseroth, 2014). Probably, by employing these tools, a temporal code of these neurons can be perturbed to have the same number but different pattern of action potentials for each encoding window. The newly created action potential patterns will carry only the original rate code information. Then the impact of these perturbations on the target neuron activity or behavior could be analyzed. However, this naïve view is a gross oversimplification of the reality as it will be shown here by analysis of the studies investigating temporal codes with the help of optogenetic tools.

In one of the first attempts to imitate neuronal code with optogenetic tools, action potential patterns were modified in dopaminergic neurons of ventral tegmental area (Tsai et al., 2009). In these neurons action potential patterns correlate with a reward-related behavior (Schultz, 2007). Under basal conditions these dopaminergic neurons spontaneously fire action potentials at ∼1 Hz and this frequency is, presumably, insufficient to activate the neuronal circuitry responsible for the development of a reward-related behavior such as place preference. Indeed, while optogenetic 1-Hz stimulation of dopaminergic neurons had no effect on behavior, 50-Hz stimulation produced a robust place preference conditioning even though the overall number of evoked action potentials was not higher during 50-Hz stimulation. These experiments showed that an increase in frequency alone is sufficient to produce the behavioral effect. Although these data favor the hypothesis that these dopaminergic neurons use rate coding, we cannot rule out entirely the contribution of temporal coding because the authors did not try to reproduce their results with the same number of action potentials elicited at the same average rate but with different spike pattern. The 50-Hz and 1-Hz stimuli differed 50-fold in duration and, in theory, the duration of a train of action potentials could be considered as a temporal code that further highlights the difficulty to discriminate between a temporal and a rate code.

In the same year two studies explicitly addressing the question of temporal coding in the brain appeared (Cardin et al., 2009; Sohal et al., 2009). Both papers investigated the same topic: the role of gamma frequency oscillations in sensory information processing in the cortex. In both cases gamma oscillations were induced artificially by employing light sensitive channels expressed in cortical fast spiking neurons. The only essential difference between two studies was the method of pyramidal neuron stimulation. In one study whisker stimulation induced responses (Cardin et al., 2009) while in another study current injections generated responses (Sohal et al., 2009). Both groups report essentially the same result. For current-injection induced responses, gamma oscillations augmented the information content (Sohal et al., 2009). It is unclear, though, what was the mechanism of this rather modest increase in the information content, by 0.19–0.24 bits or by less than 20%. Meanwhile, phase-locking of whisker stimulation to the artificially induced gamma oscillations improved the precision of action potential times, indicating an increase in the information content about whisker stimulation timing (Cardin et al., 2009). The precision of action potential timing was improved by ∼30% but it came at a price: the number of evoked action potentials was reduced by 10–50% for better-timed spikes (Cardin et al., 2009). These results indicate that temporal codes may partly contribute to sensory information processing in the cortex but do not provide a clue to what extent.

More sophisticated experiments were performed in the olfactory system, in which neurons are known to produce complex firing patterns in response to different odor presentation (Uchida et al., 2014). One such example is synchronization of mitral neurons during odor presentation. Previous research indicated that this synchronization might be important to information transfer from mitral neurons to the neurons of the posterior zone of the dorsal telencephalon, a homolog of olfactory cortex in zebrafish. Surprisingly, when mitral cell synchronization was artificially disrupted or enhanced by stimulation of light sensitive channels expressed in the mitral cell input axons, the same activity was evoked in the target neurons of the dorsal telencephalon, suggesting that the information content passed to the dorsal telencephalon was not changed (Blumhagen et al., 2011). If this result holds true, then it is one of the best arguments in the hands of the supporters of rate coding: even if we do observe synchrony in neuronal activity, it can be a side effect of rate encoding and unimportant to information processing in the brain. Nevertheless, an entirely different conclusion was reached in a study with a slightly different approach (Haddad et al., 2013). It was known that different odors generate different temporal patterns of action potentials in the mouse olfactory bulb neurons but it was unclear whether these differences in the action potential patterns are detected by the target neurons in the olfactory cortex. To answer this question, the authors imitated temporal codes of the olfactory bulb by optogenetically stimulating two locations of the olfactory bulb with varying timing between these two locations. The reported data showed that such an artificial delay between two inputs of the mouse olfactory cortex was translated into a rate code. This result suggests that such delays do encode certain stimulus features and temporal codes of the olfactory bulb neurons are transformed into rate codes in their target neurons of the olfactory cortex. This conclusion on importance of the temporal coding in the mouse olfactory bulb is indirectly supported by yet another study on the olfactory system demonstrating that mice are able to discriminate 10 ms difference between the sniff cycle phase and the time of odor presentation, clear evidence that timing is an important factor in the olfactory system (Smear et al., 2011).

It is plausible that there is no contradiction in the results of these three papers. First, different modes of temporal coding were tested, synchronization between different neurons (Blumhagen et al., 2011) and delays between two different inputs (Smear et al., 2011; Haddad et al., 2013). Second, two entirely different species were used, a zebrafish and a mouse. Finally, cortical neurons may use mostly rate coding while subcortical structures may employ mainly temporal codes (Miura et al., 2012). This hypothesis is supported by a study employing optogenetic methods that demonstrated the absence of synchronization effects on cortical responses (Histed and Maunsell, 2014). In this study it was found that in visual cortex the number of evoked action potentials but not their synchronization determines the response threshold (Histed and Maunsell, 2014). This result is related to a series of experiments designed to determine the minimal number of neurons able to impact the behavior of an animal (Houweling and Brecht, 2008; Huber et al., 2008). In these experiments both electrical and light-mediated stimulation was used. Although the first results suggested that the number of action potentials is the only factor determining the outcome of the stimulation, the follow-up study demonstrated that the pattern, i.e., the temporal code, could be of importance for the behavioral response (Doron et al., 2014).

The above-discussed papers focused on temporal codes of one neuron or a neuronal population of a single brain area. However, temporal precision of interactions between different brain areas can be regarded as a temporal code and may be as important as other forms of temporal coding. Several papers from the laboratory of Karel Svoboda investigated temporal relationships between separate brain areas by employing optogenetic tools (O’Connor et al., 2013; Guo et al., 2014). Light pulses of 100 ms or less were used to briefly interrupt neuronal activity in several brain areas during whisking. These experiments showed that there was a certain time window during which neuronal activity in a small cortical area affected behavior, confirming the importance of interaction timing between different brain areas.

Compared to overall number of papers employing optogenetic methods, there are relatively few studies that address the question presented at the outset of this paper: how important temporal codes are to information processing in the brain. Many of these papers have been discussed here. Why have so few studies addressed this apparently fundamental question of neuroscience? In the remaining part of the paper the answer to this question is sought.

Clearly, technical problems can reduce the utility of light-gated channels. The main type of light sensitive channels used for neuron excitation, channelrhodopsins, have low single channel conductance resulting into low amplitude currents of single cells even when a large number of channels are expressed in a neuron. Low amplitude current is unable to quickly charge a large neuronal membrane, resulting in a long delay between the light pulse onset and the action potential generation (Miesenbock, 2011). Nevertheless, in many cases this problem can be by-passed because the jitter of action potential times can be quite low, only a small fraction of the delay. If no repetitive stimulation at high frequencies is required, a millisecond precision still can be obtained.

Another minor technical issue is the relatively slow kinetics of channelrhodopsins that limits the maximal frequency of repetitively evoked action potentials (Miesenbock, 2011). Even though the classical channelrhodopsin-2 channels are sufficiently rapid to induce low frequency (∼40 Hz) gamma oscillations (Cardin et al., 2009), such a switching rate may fall short of the requirements for some time dependent phenomena such as LTP induction by bursts of ≥100 Hz frequency (Larson et al., 1986; Otto et al., 1991). In channelrhodopsins both the opening and the closing rates limit the speed of switching (Lin et al., 2009). An increase in light intensity speeds up channel opening rate only to a certain limit while the closing rate is largely independent of light intensity. New, faster channelorhodopsins, such as Chronos, permit switching frequencies above 100 Hz (Klapoetke et al., 2014) that may suffice for testing of most temporal codes.

In addition to technical issues, a less appreciated problem exists. In animals, especially in mammals, almost any behavior depends on multiple interacting brain areas (O’Connor et al., 2013; Guo et al., 2014). Therefore, it may be difficult to draw a connection between behavior and neuronal codes in one particular brain area because parallel circuits may obscure the impact of neuronal code perturbation. The discussed papers indicate an alternative approach. The importance of a neuronal code to information processing in the brain can be assessed by measuring the response characteristics in the target area of the neurons, the code of which was perturbed (Sohal et al., 2009; Blumhagen et al., 2011; Haddad et al., 2013). For example, to evaluate the importance of synchronization in mitral neurons to odor signal processing, the response characteristics in target neurons of dorsal telencephalon were measured after synchronization was artificially suppressed or enhanced (Blumhagen et al., 2011). The absence of any detectable change in the response features was interpreted as an indication that synchronization did not facilitate information transfer in the olfactory system. Similarly, the amplitude and precision of whisker stimulation induced responses in cortical neurons was used as a measure of the effectiveness of information transfer in the cortex (Cardin et al., 2009). Although the impact on behavior may remain the golden standard and the ultimate goal of such studies (Miesenbock, 2011), this alternative approach may yield more detailed information about the mechanisms of information processing in the brain.

This alternative method has its own caveats. The main assumption is that the measured attributes of neuronal responses contain all parameters that can be of importance to information processing in the brain. Obviously, we can never be sure about that. Nevertheless, the above-discussed studies demonstrate that this approach can yield insights that go far beyond simple correlation between the stimulus and the response. In fact, optogenetic methods may question the results obtained in such correlation-based studies by introducing a causal link between the stimulus and the response (Blumhagen et al., 2011; Haddad et al., 2013).

This short overview of the potential of optogenetic methods in investigating the importance of temporal codes to information processing in the brain cannot cover all issues related to the topic. Rather, it is an attempt to point out a direction that can facilitate such studies. It emerges from recent papers that the relationship between action potential patterns in several brain areas, representing different stages of sensory information processing, can serve as the best testing ground for optogenetic methods in probing the importance of neuronal codes to information processing in the brain. It is similar to the ‘reader-actuator’ idea that posits that two neuronal codes are meaningfully different only if the target neuronal circuitry is able to discriminate between these two codes (Buzsaki, 2010). In addition, time-dependent interaction between motor and sensory brain areas that occur during complex behaviors such as active whisking may be the most promising field for temporal code investigation (O’Connor et al., 2013; Guo et al., 2014). In order to perform such experiments, a system approach is necessary. We need more information about interactions between different brain areas contributing to action potential pattern generation; and optogenetic methods can also be of high utility (Paz et al., 2013; Mattis et al., 2014).

## Conflict of Interest Statement

The author declares that the research was conducted in the absence of any commercial or financial relationships that could be construed as a potential conflict of interest.
